# Detection of *SF3B1* p.Lys700Glu Mutation by PNA-PCR Clamping in Myelodysplastic Syndromes and Myeloproliferative Neoplasms

**DOI:** 10.3390/jcm11051267

**Published:** 2022-02-25

**Authors:** Jessica Petiti, Federico Itri, Elisabetta Signorino, Antonio Frolli, Carmen Fava, Marco Armenio, Silvia Marini, Emilia Giugliano, Marco Lo Iacono, Giuseppe Saglio, Daniela Cilloni

**Affiliations:** 1 Department of Clinical and Biological Sciences, University of Turin, 10043 Orbassano, Italy; federico.itri@unito.it (F.I.); elisabetta.signorino@unito.it (E.S.); antonio.frolli@unito.it (A.F.); carmen.fava@unito.it (C.F.); giuseppe.saglio@unito.it (G.S.); daniela.cilloni@unito.it (D.C.); 2 Department of Molecular Biotechnology and Health Sciences, University of Turin, 10126 Turin, Italy; marco.armenio@unito.it; 3 Division of Internal Medicine and Hematology, San Luigi Gonzaga Hospital, 10043 Orbassano, Italy; silvia.marini@unito.it (S.M.); e.giugliano@sanluigi.piemonte.it (E.G.)

**Keywords:** *SF3B1* p.Lys700Glu, MDS, MPN, PNA-PCR clamping

## Abstract

Mutations in *SF3B1* are found in 20% of myelodysplastic syndromes and 5–10% of myeloproliferative neoplasms, where they are considered important for diagnosis and therapy decisions. Sanger sequencing and NGS are the currently available methods to identify *SF3B1* mutations, but both are time-consuming and expensive techniques that are not practicable in most small-/medium-sized laboratories. To identify the most frequent *SF3B1* mutation, p.Lys700Glu, we developed a novel fast and cheap assay based on PNA-PCR clamping. After setting the optimal PCR conditions, the limit of detection of PNA-PCR clamping was evaluated, and the method allowed up to 0.1% of mutated *SF3B1* to be identified. Successively, PNA-PCR clamping and Sanger sequencing were used to blind test 90 DNA from patients affected by myelodysplastic syndromes and myeloproliferative neoplasms for the *SF3B1* p.Lys700Glu mutation. PNA-PCR clamping and Sanger sequencing congruently identified 75 negative and 13 positive patients. Two patients identified as positive by PNA-PCR clamping were missed by Sanger analysis. The discordant samples were analyzed by NGS, which confirmed the PNA-PCR clamping result, indicating that these samples contained the *SF3B1* p.Lys700Glu mutation. This approach could easily increase the characterization of myelodysplastic syndromes and myeloproliferative neoplasms in small-/medium-sized laboratories, and guide patients towards more appropriate therapy.

## 1. Introduction

Splicing represents the process by which introns are excised from the precursor messenger RNA (pre-mRNA) and contiguous exons are joined together [1]. In higher eukaryotes, genes undergo different splicing processes, generating different mRNA isoforms that lead to the translation of proteins with distinct functions [2]. Splicing is catalyzed by the spliceosome, a macromolecule composed of five small nuclear RNA (snRNA), each associated with proteins to form small nuclear ribonucleoproteins (snRNP).

The splicing factor 3b subunit 1 (*SF3B1*) gene is located on chromosome 2 in position 2q33.1. It encodes subunit 1 of the SF3B splice complex, a 146 kDa protein that is essential for the spliceosome function. Mutations in *SF3B1* are common in different hematological malignancies, and they were mostly found in myelodysplastic syndromes (MDS) and myeloproliferative neoplasms (MPN). MDS are clonal disorders of hematopoietic stem cells, characterized by ineffective hematopoiesis and the risk of evolving into acute myeloid leukemia (AML) [3]. Mutations in the *SF3B1* gene were found in 20% of MDS patients, particularly in 65% of MDS patients with ring sideroblasts (MDS-RS). In most cases, *SF3B1* mutations are missense and occur in heterozygosity. The most common mutation, present in 55–65% of cases, is NM_012433.4(*SF3B1*): c.2098 A>G, which leads to the substitution of lysine in glutamic acid in position 700 (p.Lys700Glu). Other sites that can be less frequently mutated are p.Glu622, p.Arg625, p.His662, p.Lys666, and p.Ile704 [4]. The strong association between mutations in *SF3B1* and the RS phenotype indicates a causal relationship, which makes this the first gene to be associated with a specific morphological feature in MDS [5]. These data allowed the WHO classification to be revised, where the RS threshold necessary for diagnosing MDS-RS in *SF3B1*-mutated patients was reduced from 15% to 5% [6]. Despite MDS-RS being characterized by a lower risk of AML evolution, patients often become resistant to erythropoietin-stimulating agents (ESA) [7] and dependent on red cell transfusions. Nowadays, a new therapeutic option for MDS-RS patients is available. Luspatercept is a recombinant fusion protein that works by binding and inhibiting the ligands of the TGFβ superfamily, interrupting TGFβ signaling, which is dysregulated in MDS [8]. The efficacy of luspatercept was evaluated in a phase II multicenter study, PACE [9], and in a phase III, double-blind, multinational randomized study, MEDALIST [10]. Both yielded positive results in terms of improved hemoglobin levels and transfusion independence. For these reasons, the *SF3B1* mutational status in MDS patients has acquired an important role in both the diagnostic and clinical settings, as it guides an important therapeutic choice.

MPN is a group of blood cancers characterized by overproduction of erythrocytes, leukocytes, or platelets in the bone marrow. In MPN, *SF3B1* was found to be mutated in approximately 5% of polycythemia vera (PV) and essential thrombocythemia (ET) patients, and 10% of myelofibrosis (MF) patients. *SF3B1* mutations in MPN do not affect survival [11], though they seem to correlate with increased thrombotic risk [12]. The initial results from a phase II study suggest clinically significant activity of luspatercept in patients with MF-associated anemia [13].

The reference technologies for the mutational analysis of the *SF3B1* gene are Sanger sequencing and next-generation sequencing (NGS). Both methods are expensive and time consuming, so evaluation of the mutational status of *SF3B1* is often not performed by small-/medium-sized laboratories.

Peptide nucleic acids (PNA), synthetic oligonucleotides with a skeleton of repeated N-(2-aminoethyl)-glycine residues joined by peptide bonds [14], can be used to evaluate the presence of specific mutations in a DNA sample by PNA-PCR clamping. The principles upon which PNA-PCR clamping is based, and some of its applications, have been amply summarized by Fouz and Appella [15]. PNA has such a high affinity for complementary DNA that even one different base prevents hybridization [16]. Furthermore, the PNA/DNA dimer cannot be amplified by DNA polymerase [17]. PNA-PCR clamping is based on the competition between a PNA probe and a primer to bind the same DNA sequence, and exploits the ability of PNA to hybridize to DNA and suppress amplification [18,19,20,21,22].

With the aim of identifying the p.Lys700Glu mutation, we developed a novel fast and cheap assay using PNA-PCR clamping. This approach could easily increase the characterization of MDS patients in small-/medium-sized laboratories, and guide them to more appropriate therapy.

## 2. Materials and Methods

### 2.1. Patient Cohort

After signing the informed consent form, 31 bone marrow (BM) and 59 peripheral blood (PB) samples were collected from 90 patients (57 MDS and 33 MPN). DNA was extracted as described by Saguna et al. [23] and quantified by spectrophotometry. Patients’ characteristics are listed in the Results section tables. In addition to the patients enrolled for screening, we tested, in duplicate (both BM and PB), the DNA of 3 mutated *SF3B1* p.Lys700Glu patients and 3 wild-type patients.

### 2.2. Cloning PCR Controls with pGEM^®^—T Easy Vector

Plasmids used as PCR positive controls were generated by amplifying *SF3B1* p.Lys700Glu and wild-type (WT) from an MDS patient with the following primers (Sigma-Aldrich, St. Louis, MO, USA): forward 5′-TGACAGGCTATGGTTC-3′ and reverse 5′-GAAACATATCCAGTTTACAT-3′. PCR products were purified by Monarch PCR & DNA Cleanup Kit (New England Biolabs, Ipswich, MA, USA) and cloned in pGEM-T Easy Vector (Promega, Milan, Italy). The sequences were verified by the capillary Sanger sequence method. All reactions were performed following the manufacturer’s instructions.

### 2.3. Sanger Sequencing for SF3B1 p.Lys700Glu Evaluation

To perform Sanger sequencing, *SF3B1* was amplified from DNA (100 ng) of patients and analyzed by sequencing with BigDye terminator v3.1 (Applied Biosystem, Foster City, CA, USA) and capillary electrophoresis on an ABI PRISM 3500XL genetic analyzer (Applied Biosystem, Foster City, CA, USA), using the primers described before. All reactions were performed following the manufacturer’s instructions. The limit of detection (LoD) of the method was previously estimated, by serial dilution experiments, to be approximately 15–20% [24].

### 2.4. PNA-PCR Clamping for SF3B1 p.Lys700Glu Evaluation

The method for the detection of the *SF3B1* p.Lys700Glu mutation by PNA-PCR clamping forecasted two PCR steps. Primers (Sigma-Aldrich, St. Louis, MO, USA) and PNA probe (Panagene, Daejeon, Korea) for *SF3B1* amplification were designed on the DNA sequence NG_032903.2.

Step 1 (pre-amplification): DNA from patients (200 ng) and control plasmids (0.01 ng) were used to amplify a small area of *SF3B1* around the c.2098 position. The primers used for the first step were the same as described before. The final volume of each reaction was 20 uL and the final concentrations of the reagents were as follows: MgCl2 [2.5 mM] (Applied Biosystem, Foster City, CA, USA), GeneAmp™ 10X PCR Buffer II [1X] (Applied Biosystem, Foster City, CA, USA), dNTP [200 nM] (Thermo Fisher Scientific, Waltham, MA, USA), *SF3B1* Fwd [50 nM], *SF3B1* Rev [50 nM], and AmpliTaq™ DNA polymerase [1U] (Applied Biosystem, Foster City, CA, USA). The amplification protocol was as follows: 94 °C × 3′, (94 °C × 30″, 55 °C × 30″, 68 °C × 40″) for 10 cycles, using a T100 thermal cycler (Bio-Rad, Hercules, CA, USA).

Step 2: 1 uL of the PCR product from the first step was used for the second step. PCR amplifications were carried out in duplicate, in the presence (TEST) or absence (CONTROL) of the PNA probe.

Primers and PNA probe sequences were as follows: forward 5′-ATGAGCAGCAGGAAG-3′, reverse 5′-GAAACATATCCAGTTTACAT-3′, and PNA probe 5′-AGCAGAAAGTTC-3′.

The final volume of the reaction was 20 uL and the final concentrations of the reagents were as follows: MgCl2 (Applied Biosystem, Foster City, CA, USA) [2.5 mM], GeneAmp™ 10X PCR Bfr II [1X] (Applied Biosystem, Foster City, CA, USA), dNTP mix [200 nM] (Thermo Fisher Scientific, Waltham, MA, USA), *SF3B1* p.Lys700Glu Fwd [100 nM], *SF3B1* p.Lys700Glu Rev [100 nM], PNA [800 nM], and AmpliTaq™ DNA polymerase [1U] (Applied Biosystem, Foster City, CA, USA).

The amplification protocol was as follows: 94 °C × 3′, (94 °C × 30″, 56 °C × 20″, 68 °C × 30″) for 33 cycles, 68 °C × 2′, using a T100 thermal cycler (Bio-Rad, Hercules, CA, USA).

After PCR amplification, each amplicon (20 uL) was mixed with DNA gel loading dye 6X (Thermo Fisher Scientific, Waltham, MA, USA), following the manufacturer’s instructions, and loaded onto 2% AgaPure™ Agarose LE (Canvax Reagents, Cordoba, Spain)-1x UltraPure™ TBE (Invitrogen, Waltham, MA, USA) gel with 5 ug/mL ethidium bromide (Thermo Fisher Scientific, Waltham, MA, USA) and run at 150 V for 30 min. The electrophoretic runs were acquired with ChemiDoc XRS+ (Biorad, Hercules, CA, USA) and analyzed with Image Lab software 4.0.1 (Biorad, Hercules, CA, USA).

### 2.5. Next-Generation Sequencing

According to the manufacturer’s recommendations, NGS libraries were obtained using the Myeloid Solution kit (Sophia Genetic, Lausanne, Switzerland) starting with the genomic DNA of the two patients with non-congruent results. The libraries were further sequenced onto a MiSeq Sequencing System (Illumina, San Diego, CA, USA) using the MiSeq Reagent Kit v3 (Illumina, San Diego, CA, USA). Data processing and BAM files were obtained by Sophia Genetics using DDMTMv5 Software (Sophia Genetic, Lausanne, Switzerland). The alignment of sequenced reads with the *SF3B1* gene in the reference genome (hg19) was visualized using the “Integrative Genomics Viewer” (IGV) (https://software.broadinstitute.org/software/igv/ (accessed on 19 July 2021); Broad Institute, University of California).

### 2.6. Statistical Analysis

Diagnostic test equivalency was checked with the McNemar statistical test. Baseline characteristics were investigated using Fisher’s exact test for categorical variables and unpaired t-test for continuous variables. WT1 expression at diagnosis was dichotomized in normal- or high-expression group, as indicated in the literature [25] (BM: 90 *WT1*/10^4^ *ABL1* copies; PB: 10 *WT1*/10^4^ *ABL1* copies). Statistical analyses were performed using GraphPad Prism 7 statistical software. All the analyses with a p-value less than or equal to 0.05 were indicated as significant.

## 3. Results

### 3.1. Detection of SF3B1 p.Lys700Glu Mutation by PNA-PCR Clamping

**Figure 1:**
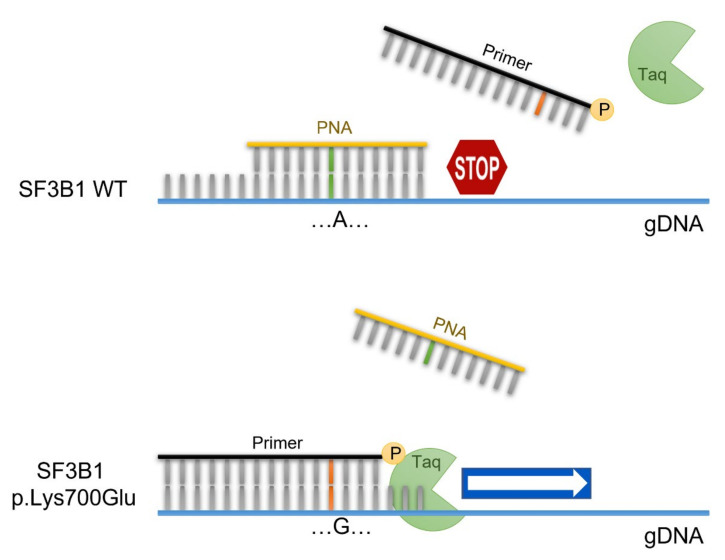
PNA-PCR clamping experimental design. Amplification of *SF3B1* is performed in presence of the PNA probe (**yellow**), designed on the WT sequence, and the primer competitor (**black**), designed on the mutated sequence. In these conditions, the PCR of the WT sequence is inhibited by the hybridization of PNA/DNA. In contrast, in the presence of the *SF3B1* p.Lys700Glu genotype, the primer/DNA duplex allows the amplification of the target sequence.

The PNA-PCR clamping method is based on direct competition between the PNA probe and one of the primers to bind the complementary genomic DNA sequence (gDNA). If the *SF3B1* gene is WT, the PNA probe, drawn on the WT sequence, pairs perfectly with the gDNA and inhibits its amplification. On the contrary, the presence of the p.Lys700Glu mutation favors the bond between the gDNA and the primer, which is complementary to the mutated sequence, making mutated gDNA amplification possible (Figure 1).

**Figure 2:**
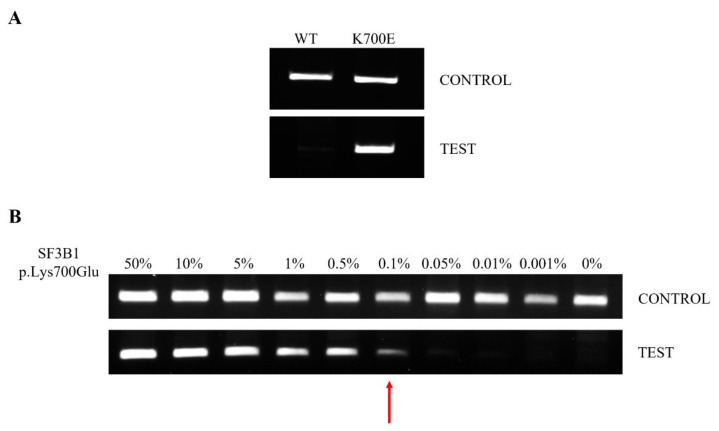
Electrophoretic runs of PCR reactions: each amplicon was loaded on 2% agarose-TBE 1× gel with 5 μg/mL EtBr and run at 150 V for 30 min. PNA-PCR clamping for *SF3B1* was carried out in the absence (CONTROL) and presence (TEST) of the PNA probe. (**A**) Example of PNA-PCR clamping in DNA from WT and p.Lys700Glu-mutated patients. CONTROL PCR represents an internal control and DNA must always be amplified. TEST PCR returns the result about the mutational status of *SF3B1*; if the patient is *SF3B1* WT, the gDNA is not amplified, while if the patient is *SF3B1* p.Lys700Glu, the gDNA is amplified. (**B**) PNA-PCR clamping LoD was assessed by mixing pGEMT-*SF3B1* p.Lys700Glu and pGEMT-*SF3B1* WT plasmids at different ratios in the same PCR reaction. Dilutions were as follows: 50, 10, 5, 1, 0.5, 0.1, 0.05, 0.05, 0,01, 0.001 and 0% pGEMT-*SF3B1* p.Lys700Glu, all brought to 100% with the respective amount of pGEMT-*SF3B1* WT template. The percentage of the mutated template is indicated above each amplicon. The red arrow indicates the LoD of the PNA-PCR clamping method.

This method involves the following two PCR steps: The first consists of pre-amplification for a short amount of time, to increase the specificity of the test, avoiding the non-specific amplification of other fragments by second-step primers. The second step consists of the mutational test, and is carried out in duplicate, in the presence (TEST) or absence (CONTROL) of the PNA probe. In CONTROL PCR, the DNA must always be amplified. Otherwise, the result will not be valid, and the assay will have to be repeated. If the CONTROL PCR is valid, TEST PCR returns the result about the mutational status of *SF3B1*; the patient will be *SF3B1* WT if the DNA is not amplified, while the patient will be *SF3B1* p.Lys700Glu if the DNA is amplified (Figure 2A).

The LoD of the method was evaluated by mixing, at different concentrations, the pGEMT-*SF3B1* p.Lys700Glu and pGEMT-*SF3B1* WT plasmids. The dilutions used were as follows: 50, 10, 5, 1, 0.5, 0.1, 0.01, 0.001 and 0% of pGEMT-*SF3B1* p.Lys700Glu in pGEMT-*SF3B1* WT. The method showed a very low LoD, allowing us to identify up to 0.1% of mutated *SF3B1* (Figure 2B).

### 3.2. Comparison of Sanger Sequencing and PNA-PCR Clamping for Detection of SF3B1 p.Lys700Glu

Before patient screening was started, we tested, in duplicate (both BM and PB), the DNA of two mutated *SF3B1* p.Lys700Glu and three wild-type patients with PNA-PCR clamping. No difference was observed between the results obtained for the BM and PB samples. Then, PNA-PCR clamping and Sanger sequencing were used to blind test 90 DNA from 57 MDS and 33 MPN patients for the *SF3B1* p.Lys700Glu mutation. PNA-PCR clamping and Sanger sequencing congruently identified 75 negative and 13 positive samples, while two MDS patients identified as positive by PNA-PCR clamping were missed by Sanger analysis (Table 1).

**Figure 3:**
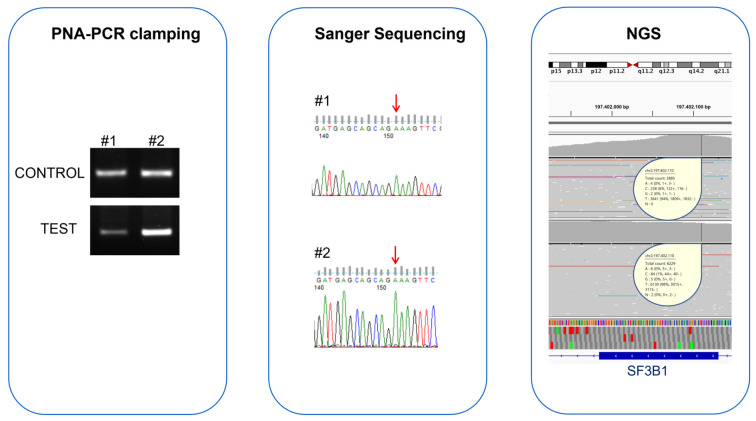
Comparison of PNA-PCR clamping, Sanger sequencing, and NGS for the evaluation of *SF3B1* status in two MDS patients with contrasting results. The electrophoretic runs of PNA-PCR clamping, carried out in the absence (CONTROL) and presence (TEST) of the PNA probe, showed amplification in both the samples, indicating the presence of the *SF3B1* p.Lys700Glu mutation in these patients. In contrast, Sanger sequencing chromatograms showed the presence of only one allele (red arrows); the nucleotide “A” indicates that both patients were WT for *SF3B1* p.Lys700Glu mutation. NGS confirmed the PNA-PCR clamping result, indicating that these samples contained *SF3B1* p.Lys700Glu with mutation percentages of 1.3% and 6.1%, respectively. Both samples had mutation percentages lower than the Sanger sequencing LoD, but they were detected by PNA-PCR clamping methodology.

The agreement between Sanger sequencing and PNA-PCR clamping was confirmed by McNemar’s test, which did not highlight differences in the proportion of disagreement data. To understand if the discordant samples were PNA-PCR clamping false-positives or Sanger sequencing false-negatives, we analyzed them with NGS. NGS confirmed the PNA-PCR clamping result, indicating that these samples possessed *SF3B1* p.Lys700Glu with mutation percentages of 1.3% and 6.1%, respectively (Figure 3).

Interestingly, to identify enough mutated sequences to increase confidence in the results, it is necessary to perform deep sequencing with more than a thousand reads for each patient, even with NGS. In particular, for the first patient, we only identified 84 mutated reads out of the total 6229, while 238 out of 3885 total reads were mutated for the second patient.

### 3.3. Prevalence of SF3B1 p.Lys700Glu in MDS and MPN Samples

In our cohort of patients, we found the *SF3B1* p.Lys700Glu mutation in 24.5% (14/57) of MDS patients and 3% (1/33) of MPN patients (Table 2).

For the MDS samples, we compared the baseline characteristics of mutated and WT patients. We did not find any significant correlations between the *SF3B1* p.Lys700Glu mutation and age, sex, or IPSS-R. Fisher’s exact test confirmed the significant strong association between the p.Lys700Glu mutation and the RS phenotype (*p* < 0.0001). Indeed, 85.7% of mutated patients were classified as MDS-RS, while only 14.3% were MDS-MLD. In the *SF3B1* p.Lys700Glu WT group, only 9.3% of the patients showed RS. Furthermore, by dichotomizing the samples, based on *WT1* expression at diagnosis in the normal- or high-expression group, as indicated in the literature [25], we reported that the p.Lys700Glu mutation correlates with normal *WT1* expression at diagnosis (*p* = 0.022). Because the expression of *WT1* at normal levels is an indicator of a good prognosis, this result corroborates the role of *SF3B1* status as a good prognosis marker.

Regarding the MPN cohort, we only found one mutated sample. The patient was a 69-year-old male, with an MF secondary to ET and intolerant to ruxolitinib treatment. The patient had a concomitant *JAK2* V617F mutation and a deletion on chromosome 20q.

## 4. Discussion

Molecular characterization has become mandatory in the setting of myelodysplastic syndromes and myeloproliferative disorders. The 2016 WHO classification recognized MDS with ring sideroblasts as an entity defined by the presence of more than 15% of ring sideroblasts or more than 5% with the *SF3B1* mutation.

MDS with the RS phenotype are associated with a shorter median duration of response to ESA, and with fibrosis [7,26]. For these reasons, the assessment of the mutational status of *SF3B1* has become relevant not only for a correct diagnosis, but also for offering the possibility of an effective therapy. The most used methods for identifying *SF3B1* mutations are Sanger sequencing and NGS. The main limit of Sanger sequencing is its low sensitivity, which means that it is unable to detect small clones. By contrast, NGS is much more sensitive, but is burdened by rather long times and fairly high costs. Although the search for mutations with prognostic significance should be sought in all patients for a correct diagnostic classification, in “real life”, the search for mutations with NGS is reserved for younger patients with therapeutic perspectives. Frequently, the patient chosen for supportive therapy only, including transfusions, is not molecularly typed in order to prevent overloading diagnostic laboratories. In this study, we designed and validated a new technology that is sensitive, fast, and cheap to identify the *SF3B1* p.Lys700Glu mutation based on PNA-PCR clamping, and we compared this technique with Sanger sequencing. PNA-PCR clamping rapidly identified the *SF3B1* p.Lys700Glu mutation in DNA samples. The PNA-PCR clamping technique showed high efficiency, sensitivity, and specificity, and it was faster and cheaper than Sanger sequencing. PNA-PCR clamping showed an LoD of about 0.1% of a mutation that was extrapolated in vitro by serial dilutions of mutated plasmids, and subsequently confirmed in DNA samples. This limit of detection is markedly lower than the Sanger method, which is estimated, in the literature, to be 15–20% [24]. Indeed, in our analysis, Sanger sequencing showed two cases of false-negatives, while the mutation was detected by PNA-PCR clamping, in agreement with NGS analysis. Furthermore, even with NGS, it is necessary to obtain a high sequence depth to obtain confident results, a depth that is not always guaranteed by routine diagnostic conditions. Malcovati et al. reported that the median variant allele frequency (VAF) for the *SF3B1* mutations is 39.5% in MDS patients, with a high variable range from 5.4 to 70.3% [5]. The low LoD that our assay showed can easily cover the entire range of VAF described in the literature, minimizing the false-negative results of Sanger (LoD of about 15–20%) and/or high-resolution melting (HRM) analysis (LoD of about 10%) [27]. Few data are currently available about the role of small mutated *SF3B1* clones, but it is known that *SF3B1* mutations can propagate from hematopoietic stem cells to their progeny [28]. Furthermore, xenograft mice, in which *SF3B1* subclones were <5% of the total grafted cells, also developed an MDS phenotype [28]. Although a clear prognostic role of small *SF3B1*-mutated clones is not known to date, having a diagnostic assay capable of identifying them may still be useful for a “watch and wait” approach, and for an early therapeutic decision.

A possible limitation of our test is the evaluation of only one of the *SF3B1* mutations, the most frequent one. Although the p.Lys700Glu mutation covers only 55–65% of all *SF3B1* mutations, the remaining 35–45% is characterized by a multitude of different mutations, including p.Glu622Asp, p.Tyr623Cys, p.Arg625His/Gly/Cys/Leu, p.Asn626Asp, p.His662Gln/Asp, p.Lys666Glu/Asn/Arg/Thr, and p.Ile704Val/Asn, each present with a very low frequency (<5%). From a prognostic point of view, the available data are very conflicting. Malcovalti et al. highlight the positive independent prognostic value of *SF3B1* mutations in sideroblastic categories only, while, in MDS with excess blasts, the mutation did not have a significant effect on survival and risk of disease progression [29,30]. By contrast, Dalton et al. report that the p.Lys666Asn hotspot is associated with MDS with excess blasts and increased progression to acute myeloid leukemia [31]. More recently, Kanagal-Shamanna et al. found that only the p.Lys700Glu mutation independently predicts better overall survival in MDS, suggesting that the identification of this *SF3B1* mutation type is important for risk stratification [32]. In this paper, we demonstrated the feasibility and utility of the PNA-PCR clamping approach in the identification of the most frequent *SF3B1* mutations. For a more complete analysis, it will be necessary to improve our system by including the mutations in the p.Lys666 position to cover the majority of *SF3B1* mutations.

Compared with other currently available methods, such as Sanger sequencing and HRM analysis, PNA-PCR clamping has been shown to have a better LoD and a lower cost, both in terms of reagents and instrumentation. Although the other two techniques cited allow other *SF3B1* mutations to also be evaluated, PNA-PCR clamping can be easily implemented to evaluate other hotspots, without significantly affecting the cost and timing of execution, which remain lower than Sanger sequencing or HRM analysis.

In addition, we confirmed the role of mutated *SF3B1* as a molecular biomarker of the MDS-RS subgroup [12,33], and we showed a correlation between the mutational status of *SF3B1* and *WT1* expression. Indeed, MDS patients with a mutated *SF3B1* genotype showed lower *WT1* expression values than the WT counterpart. Because the expression of *WT1* at normal levels is an indicator of a good prognosis, this result corroborates the role of *SF3B1* status as a good prognosis marker [5,34,35,36].

## 5. Conclusions

Considering the accuracy and sensitivity of PNA-PCR clamping, its rapidity, and its cost, we propose this method for basal molecular typing of MDS. Although it cannot replace NGS, PNA-PCR clamping will allow a percentage of patients to have a molecular assessment, which, although incomplete, offers the possibility of identifying an effective therapy.

## Figures and Tables

**Table 1:** PNA-PCR clamping and Sanger sequencing results.

		PNA-PCR Clamping		
		Negative	Positive	TOT
**Sanger seq**	**Negative**	75	2	77
	**Positive**	0	13	13
	**TOT**	75	15	90

**Table 2:** MDS patients and MPN patients’ characteristics.

Characteristics (MDS)	*SF3B1* WT		*SF3B1* p.Lys700Glu		*p* Value
	(*n* = 43; 75.4%)		(*n* = 14; 24.6%)		
	*n*	%	*n*	%	
Age (years)					ns
Median (range)	76 (31–94)		74 (51–83)		
Sex					ns
Male	28	65.1	7	50.0	
Female	15	34.9	7	50.0	
WHO Classification					****
RS	4	9.3	12	85.7	
SLD	9	20.9	0	0.0	
MLD	14	32.6	2	14.3	
5q-	3	7.0	0	0.0	
EB1/EB2	8	18.6	0	0.0	
na	5	11.6	0	0.0	
IPSS-R					ns
very low/low	24	55.8	11	78.6	
intermediate	10	23.3	3	21.4	
high/very high	6	14.0	0	0.0	
na	3	7.0	0	0.0	
*WT1*/10^4^*ABL1* copies					*
normal	13	30.2	10	71.4	
high	23	53.5	3	21.4	
na	7	16.3	1	7.1	
**Characteristics (MPN)**			***n* = 33**		
			*n*		**%**
Age (years)					
Median (range)			73 (52–88)		
Sex					
Male			14		42.4
Female			19		57.6
*SF3B1* status					
WT			32		97.0
p.Lys700Glu			1		3.0
MPN type					
PV			6		18.2
ET			0		0.0
MF			27		81.8
MF evolution					
PMF			11		40.7
PPV-MF			8		29.6
PET-MF			8		29.6
*WT1*/10^4^*ABL1* copies					
normal			5		15.2
high			27		81.8
na			1		3.0

Abbreviations: MDS, myelodysplastic syndromes; wt, wild-type; *n*, number; RS, ring sideroblast; SLD, single lineage dysplasia; MLD, multilineage dysplasia; EB, excess blasts; na, not available; MPN, myeloproliferative neoplasms; PV, polycythemia vera; ET, essential thrombocythemia; MF, myelofibrosis; PMF, primary MF; PPV, post-PV; PET, post-ET. All the analysis with confidence level major of 95% are indicated like significant and marked as followed: * *p* ≤ 0.05; **** *p* ≤ 0.0001; ns, not significant.

## Data Availability

Data are contained within the article.
